# Coronary artery haematoma after primary percutaneous coronary intervention: late, trans-stent, retrograde progression

**DOI:** 10.1093/ehjcr/ytae049

**Published:** 2024-01-25

**Authors:** Yoshihiro Harano, Yoshiaki Kawase, Hitoshi Matsuo

**Affiliations:** Department of Cardiovascular Medicines, Gifu Heart Centre, 4-14-4 Yabutaminami, Gifu 500-8384, Japan; Department of Cardiovascular Medicines, Gifu Heart Centre, 4-14-4 Yabutaminami, Gifu 500-8384, Japan; Department of Cardiovascular Medicines, Gifu Heart Centre, 4-14-4 Yabutaminami, Gifu 500-8384, Japan

One of the common complications of percutaneous coronary intervention (PCI) is iatrogenic coronary intramural haematoma (CIH), which sometimes worsens in the days following the PCI.^1^

A 73-year-old woman was transferred to our hospital due to unstable angina. Initial coronary angiography showed a severe lesion in the middle of the left anterior descending artery (LAD), and intravascular ultrasound study (IVUS) revealed a low-echoic plaque but no haematoma at the lesion (*Figure 1A*). Emergent PCI was performed by implantation of a 2.5 × 38 mm drug-eluting stent (DES) followed by post-dilation with a 3.0 × 12 mm non-compliant balloon at the stent proximal zone. The presence of CIH at the distal end of the stent was subsequently demonstrated on IVUS imaging (*Figure 1B*). Additional stenting was not performed as a subsequent IVUS conducted 15 min later showed no progression of the CIH. Due to the lack of complete recovery of the chest discomfort, the patient was closely followed in our hospital. On Day 7, the patient’s chest pain suddenly worsened and the electrocardiogram showed newly developed ST-segment elevation in the inferior leads. Emergent coronary angiography and IVUS demonstrated that the CIH had surprisingly progressed retrogradely beyond the implanted DES to the left main trunk (LMT) and the left circumflex artery (LCX) (*Figure 1C*). A bailout PCI was performed by covering the haematoma using a T-stent method: one 4.0 × 21 mm DES from the LMT to the proximal LAD and the other 3.5 × 18 mm DES in the proximal LCX (*Figure 1D*). Follow-up coronary computed tomographic angiography on the following day revealed no further progression of CIH.

**Figure 1:**
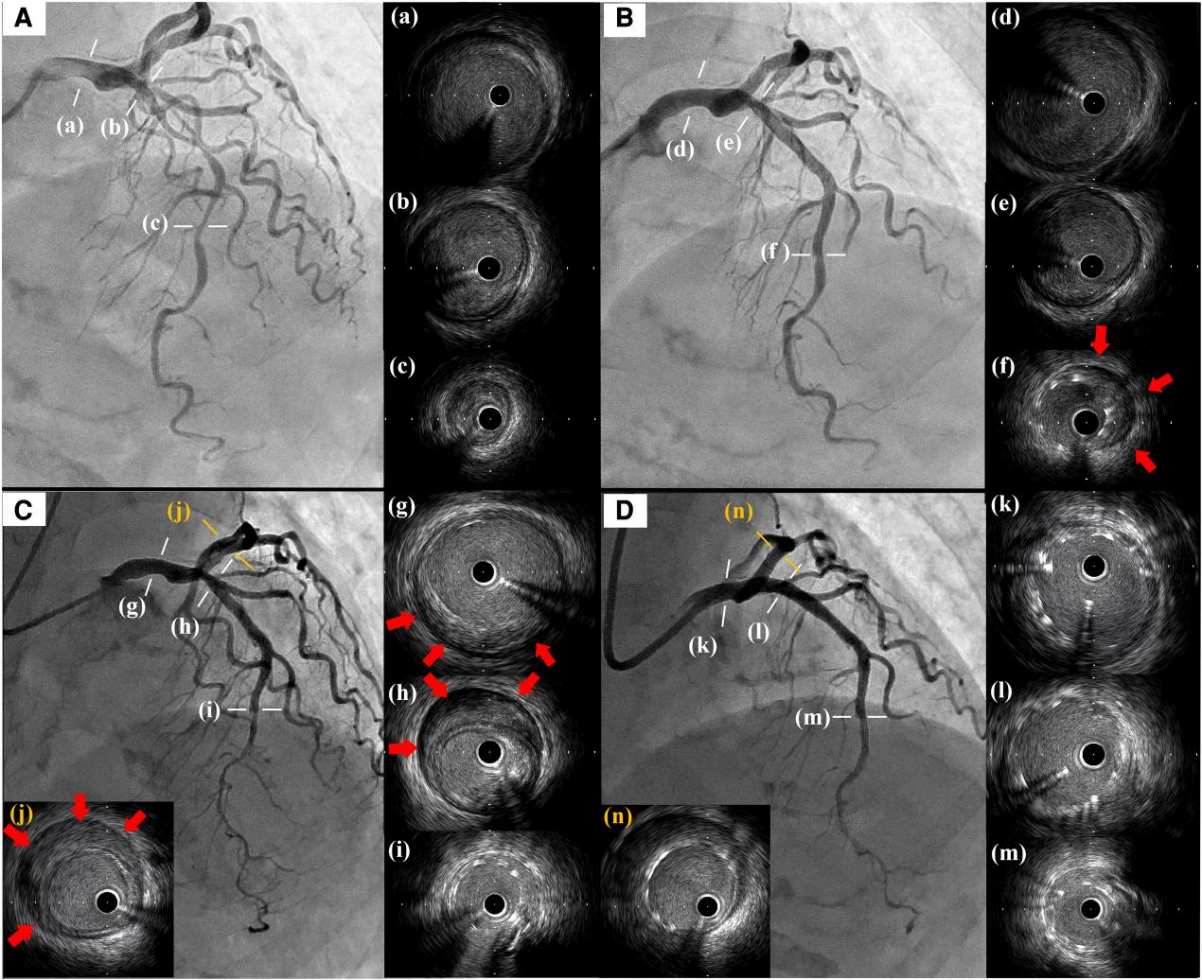
(*A*) Initial left coronary angiography from a straight cranial view and images of IVUS (a–c). IVUS demonstrated no haematoma but low-echoic plaque. (*B*) Post-primary PCI. IVUS revealed haematoma in the stent distal (red arrows) but not in the proximal vessel (d–f). (*C*) Emergent coronary angiography and IVUS on day seven. The previous haematoma extended to the LMT through the LAD (g–i) and even to the proximal LCX (j). Red arrows indicated the newly observed haematoma. (*D*) Final angiography and IVUS after the bailout PCI. Additional stents were implanted, covering the LMT to the LAD (k–m) and the LCX (n) using the T-stent methods. IVUS, intravascular ultrasound study; PCI, percutaneous coronary intervention; LMT, left main trunk; LAD, left anterior descending artery; LCX, left circumflex artery.

Iatrogenic CIH occurred in 6.7% of patients after PCI and sometimes worsened in the days or weeks following the intervention.^1^ In this case report, we demonstrate how the haematoma can advance retrogradely even in the presence of an implanted stent.

**Consent:** The authors confirm that written consent for submission and publication of this case report, including images and associated text, has been obtained from the patient according to the COPE guidelines.

**Funding:** None.

## Data Availability

The data underlying this article will be shared on reasonable request to the corresponding author.
